# Comparison of 15–20 mmHg versus 20–30 mmHg Compression Stockings in Reducing Occupational Oedema in Standing and Seated Healthy Individuals

**DOI:** 10.1155/2018/2053985

**Published:** 2018-10-01

**Authors:** Cleusa Ema Quilici Belczak, José Maria Pereira de Godoy, Amélia Cristina Seidel, Sergio Belczak, Rubiana Neves Ramos, Roberto Augusto Caffaro

**Affiliations:** ^1^João Belczak Vascular Center, Maringá, PR, Brazil; ^2^Department of Cardiology and Cardiovascular Surgery, Medicine School of São José do Rio Preto and Research CNPq (National Council for Research and Development ), SP, Brazil, Brazil; ^3^Medicine Course of Maringá State University, Maringá, PR, Brazil; ^4^Medicine Course of São Camilo University Center, São Paulo, SP, Brazil; ^5^Medicine Course of UNICESUMAR, Maringá, PR, Brazil; ^6^Division of Vascular Surgery, Department of Surgery, Medicine School of the Santa Casa de Misericórdia de São Paulo, SP, Brazil

## Abstract

**Background:**

Elastic compression stockings (ECS) are effective in preventing and reducing occupational edema (OE), but the optimal pressure according to the prevalent working position during the day is still controversial.

**Objective:**

To compare the effectiveness of ECS with different pressures (15–20 mmHg or 20–30 mmHg) for reducing OE in individuals working in different prolonged postures.

**Methods:**

This cross-sectional study comprised 116 lower limbs of 58 individuals divided into three groups according to their prevalent postures over the day (sitting, standing, or combination). Volumetric measurements were taken at the beginning and at the end of three consecutive days. On the first day, individuals did not use compression stockings; on the second and third days, they used, respectively, 15–20 mmHg and 20–30 mmHg knee-length stockings. Differences between morning and evening volumes (measured edema) were calculated, compared, and correlated.

**Results:**

Volumetric variations were significantly lower on the second compared to the first day when individuals in all three groups used 15–20 mmHg compression stockings (p-value < 0.001). Measurements were even lower when they used 20–30 mmHg stockings: this decrease was more significant for the sitting (p-value < 0.001) than the standing (p-value < 0.05) and combined groups (p-value < 0.05). Reduction of measured edema was more significant in individuals working in a prolonged seated position. No significant difference was found only on comparing sitting and standing groups after the use of the 15–20 mmHg compression stockings.

**Conclusions:**

The use of ECS over a working day reduces OE in prolonged sitting, standing, and combined positions, with the reductions being greater with the higher pressure.

## 1. Introduction

Occupational edema (OE) of the lower limbs (LL) has been associated with venous diseases since 1960 [1–4]. However, some authors reported that there is inconsistent data related to this association, arguing that previous studies had included individuals without venous diseases as such, but with some suggestive symptoms, especially the feeling of heaviness and swelling [5–7]. Now it is clear that occupational postures adopted for long periods over a working day result in measurably increased volumes of the LL even in healthy individuals [8–10], and that this increase is more intense at the end of the morning compared to the gain observed at the end of afternoon [11, 12]. There has also recently been a suggestion that greater OE is more common in individuals who work for longer stretches in the seated position than in those who stand for long periods [13].

The fact is that OE is uncomfortable; it causes a feeling of heaviness and tiredness and can lead to reduced productivity, increased work absenteeism, and poor quality of life, as well as being perhaps one of the first manifestations of decompensation of the venous and lymphatic systems [12], in particular in individuals with higher body mass indexes [14].

Although walking, muscle [15], and water exercises [16] help to reduce OE, the best preventive measurements are achieved with the use of elastic compression stockings (ECS) [17–25], even when they are used for only half a working day [22]. For the most beneficial effects of ECS, however, it is necessary to establish the best compression pressure, since consensual recommendations still refer more to chronic lymphatic and venous diseases [24]. In a systematic review, Amsler et al. [21] concluded that 10-15 mmHg compression is effective in preventing OE and patients' complaints; less pressure is ineffective and higher pressures may be of no additional benefit.

However, it is not yet clear whether the same compression would be effective for OE in individuals subjected to different prevalent postures over the working day. Partsch et al. [20] reported that calf-length ECS with a pressure range between 11 and 21 mmHg can reduce or totally prevent OE in individuals with a profession requiring long periods of sitting or standing. The effectiveness of light ECS (10-15 mmHg) to reduce the formation of edema after prolonged sitting and standing was confirmed by others, according to a very complete review carried out for a consensus on indications for compression therapy in venous and lymphatic diseases [26].

The objective of this study was to compare the effectiveness of ECS with different pressures (15-20 mmHg and 20-30 mmHg) in individuals working in different prolonged postures (sitting, standing, and combination).

## 2. Materials and Methods

This observational cross-sectional study was performed at the João Belczak Vascular Center, Maringá, PR, Brazil, between 2014 and 2015. Individuals, who accepted to participate in this study after responding to an invitation sent to local beauty salons and to a private hospital located in that city, were selected for the study.

Clinical history and clinical examinations were carried out by a vascular clinician in all individuals after they had consented to be included in the study. Exclusion criteria included the presence of ischemia, hypertension, diabetes, current use of diuretic or hormonal medications, systemic LL edema resulting from congestive heart disease, renal or hepatic insufficiency, myxedema, traumatic or rheumatic articular diseases, lymphedema, daily use of compression stockings, and any type of skin lesion.

Fifty-eight professionally active individuals without symptoms of venous insufficiency or just with telangiectasia (C0 and C1 in the CEAP classification) were included in the study. According to their prevalent occupational positions during the working day, they were divided into three study groups: seated position (SIT), standing position (STD), and a combination of sitting, standing, and walking in similar proportions of time (COMB).

Volumetric measurements (milliliters) using the water displacement technique were taken of both legs of each individual always by the same physical therapist at the beginning and at the end of each of three consecutive working days. This technique is the gold standard, but it requires an experienced professional so that the results are accurate and reproducible. Some technical precautions should be taken, such as the positioning of the leg on the side of the device to prevent movement that can cause excessive water displacement affecting the results. Volumetric (**milliliters**) measures are taken on the first day (without stockings) at morning and at evening in the three posture groups. On the first day, individuals did not use compression stockings at all. On the second and third days, they used stockings with pressures of 15 to 20 mmHg and 20 to 30 mmHg (Medi ® Brazil), respectively. Volumes were measured at the same time every day (7:00 am and 7:00 pm), and climatic conditions were very similar over the three days. Differences between morning and evening volumes were calculated.

The Wilcoxon, Mann-Whitney, Friedman plus Dunn multiple comparisons and Kruskal-Wallis tests, Student's t-test, Qui-square Test, Anova, and Pearson's* r* correlation were used for statistical analysis. An alpha error of 5% (p-value ≤ 0.05) was considered significant.

## 3. Results

Characterization of these groups is presented in Table 1. The Body Mass Index (BMI) was significantly higher in the SIT group compared to the COMB group. Groups were homogeneous regarding gender, age, and race. The SIT group was composed of five manicurists, six office assistants, and nine secretaries; all 18 individuals of the STD group were hairdressers and there were five cleaners, one doctor, two physical therapists, and 12 secretaries in the COMB group.

Preliminary analysis of our data did not identify any significant differences between right and left legs in the three groups of occupational postures or between the three conditions (without compression stockings and with 15-20 mmHg and 20-30 mmHg ECS). Thus, an analysis was conducted considering the 40 LL in the SIT group, 36 LL in the STD group, and 40 LL in the COMB group.

**Figure 1:**
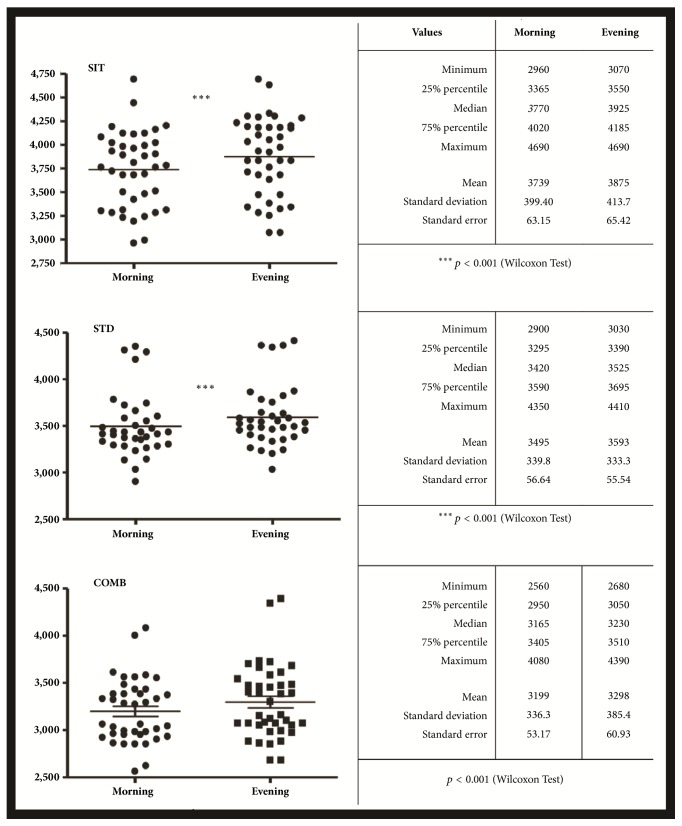
Volumetric (milliliters) measures taken on the first day (without stockings) at morning and at evening in the three posture groups.

On the first day, OE was found in all three groups when not using compression stockings. Mean volumetric measurements taken in morning and evening on the first day (without stockings) were significantly higher in the SIT group compared to the other two groups, as well as between the STD and the COMB group (Figure 1).

**Figure 2:**
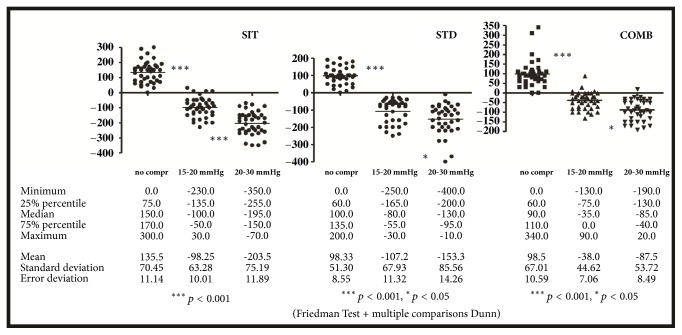
Volumetric differences between morning and evening measures taken without compression stockings (first day) and after a working day using 15-20 mmHg (second day) or 20-30 mmHg (third day) compression stockings.

Edema variations and thus edema were significantly lower on the second day when individuals used 15-20 mmHg compression stockings compared to the first day without stockings (p-value < 0.001) in all three groups. With the use of 20-30 mmHg stockings, measurements were even lower: this decrease was more significant in the SIT group (p-value < 0.001) than in STD (p-value < 0.05) and COMB (p-value < 0.05) groups (Figure 2).

**Figure 3:**
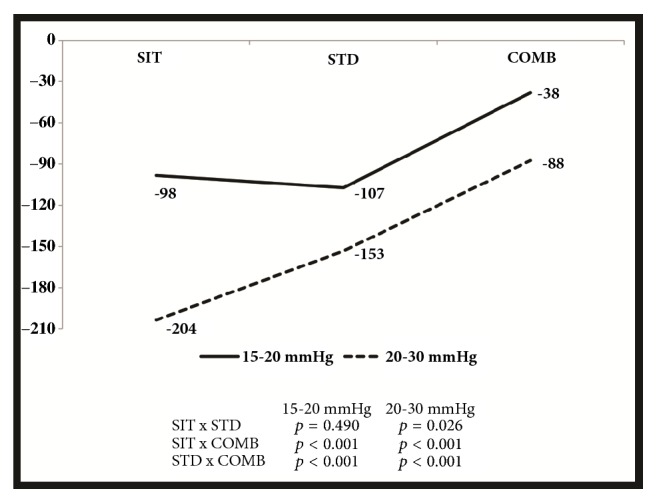
Graphic representation of the mean volumetric variations after a working day using 15-20 mmHg (second day) or 20-30 mmHg (third day) compression stockings.

The volumetric variations after the use of 15-20 mmHg compression stockings for a working day did not differ between the three groups (Figure 3).

## 4. Discussion

Results of this study firstly confirmed the presence of measurable OE consequent to prolonged sitting, standing, or combined postures over a working day, as well as the efficiency of the ECS to significantly reduce this OE. The reduction of the OE was already significant on the second day of the study, when individuals of the three posture groups used 15-20 mmHg ECS; the reduction became even more significant with ECS with the higher pressures (20-30 mmHg) used on the third day.

On the first day, when individuals did not use ECS, the mean volumetric measurements taken at the end of the working day were significantly higher than those in the morning for all posture groups. The highest measurements were presented by the SIT group, followed by the STD and the COMB groups, both in the morning and in the evening. We believe that the immobility of the LL in the prolonged seated position results in worse phlebologic conditions, since the lack of movements of the ankles negatively affects the functioning of the calf pump. This finding suggests that in the prescription of a medical elastic stocking consider the occupation of each patient in which the seated position suggests need for a half of more compression. It is warned that the patient's adherence to the treatment is fundamental and it is the one that determines the therapeutic success and thus suggests discussing the benefits and discomforts with regard to the type of stocking to be individually prescribed. Although with prolonged standing, the LL are not moved much; they are not as immobile as in the SIT group as movement is needed to maintain the standing position, and thus the calf-muscle pumps are activated to some extent promoting reductions of the distal venous pressure. Furthermore, we can observe empirically that individuals who work in the seated position over seven or eight hours a day are those who present more complaints compatible with venous insufficiency.

In fact, individuals without vascular disease present pressures of 90 mmHg when standing with this pressure decreasing to 30 mmHg when walking. On the other hand, in the seated position, the pressure is about 52 mmHg with this elevated pressure being constant while the individual remains seated, thereby explaining the tendency of accumulating more edema and developing symptoms of chronic venous insufficiency.

On the other hand, individuals working for prolonged periods sitting or standing or both presented similar reductions in the LL mean volumes when using 15-20 mmHg ECS. However, when using 20-30 mmHg ECS, this reduction was significantly higher in the SIT group compared to the other two groups. This means that ECS with higher pressures seem to be more appropriate for individuals who sit for most of the working day.

Studies on the optimal compression pressure to reduce edema generally refer to chronic venous diseases [24–27] or, when analyzing OE, they report on sitting and standing as only one category of prolonged working posture [20, 21]. This could be the reason that light ECS (10-15 mmHg) were reported as providing beneficial effects related to the reduction in the formation of edema after prolonged sitting and standing [20, 25]. The suggestion that compression higher than 10-15 mmHg may be of no additional benefit deserves to be reviewed, since the studies based on this conclusion refer predominantly to prolonged standing [21].

Because of the difficulty in selecting healthy individuals willing to participate in this kind of study, our sample was not randomized, and demographic/clinical variables could not be controlled, thus imposing an important limitation on this study. Even taking into account the homogeneity of the groups regarding gender, ethnic group, and age, we have to consider that individuals allocated to the SIT group had a significantly higher mean BMI than the other groups, which maybe justifies the higher volumetric measurements in this group, although the association between LL edema and obesity is still controversial in healthy individuals [14]. The highest BMIs referred to individuals in the STD and COM groups, indicating that this variable may not have had an effect on the findings.

In spite of these limitations, our objective was very specific: to verify whether ECS with different pressures would reduce OE in the same way in individuals subjected to prolonged sitting, standing, and combined postures. The findings of this study confirmed that the use of ECS over the working day reduces OE in the different conditions (sitting, standing, and combination) and evidenced that 20-30 mmHg ECS are more effective, especially for individuals working in the prolonged seated position.

Another aspect to be considered is the physiological edema that can be observed in the CEAP C0 and C1e even C2. However, with significant temperature variations, in the heat period the number of symptomatic patients with pain and edema increases greatly. In the personal experience in the city of São José do Rio Preto, Brazil, in the winter period there are almost no symptomatic patients with edema with CEAP C2 or lower. Thus, the symptoms associated with chronic venous insufficiency are multifactorial and of an impossible distinction of what is venous, interference of temperature, gravitational posture, and other changes such as flat feet. We believe that symptomatic patients with significant venous reflux may progress to chronic venous disease. However, symptomatic patients with no reflux may not evolve to chronic venous disease. What differentiates in relation to CEAP C3 is an association of greater severity of the venous disease in relation to the minor APCs.

## 5. Conclusions

The different occupational positions, sitting, standing, and a combination of both, affect the evolution of LL edema during the course of the day. The use of therapeutic elastic stockings is a manner to protect against the progress of edema, where higher compression provides greater protection.

## Figures and Tables

**Table 1:** Characterization of 60 professionally active individuals according to their prevalent occupational postures along a working day.

Parameters		Groups of prevalent occupational posture			
		SIT	STD	COMB	*p*-value
Gender (%)	Female	75.0%	88.9%	100.0%	
	Male	25.0%	11.1%	-	0.132^1^

Ethnic	Black	15.0%	5.6%	5.0%	
group (%)	Mulatto	25.0%	27.8%	30.0%	
	White	55.0%	55.5%	65.0%	
	Yellow	5.0%	11.1%	-	0.518^1^

Age (years)	Range	23 – 63	24 – 63	23 – 64	
	Mean ± Standard deviation	41.8 ± 12.1	39.1± 11.9	44.1 ± 11.8	> 0.05^2a^
	Median	42	37	44	

BMI	Range	22.8 – 34.0	17.9 – 36.1	15.4 – 34.7	
	Mean ± Standard deviation	27.1 ± 3.0	26.1 ± 4.3	24.5 ± 3.8	<0.05^2b^
	Median	26	25	24	

(1) Qui-square test; (2a,b) Anova; (2b) SIT *versus* COMB: *p* = 0.020 (Student's *t*-test).

## Data Availability

The data used to support the findings of this study are included within the supplementary information files.

## Supplementary Materials

Supplementary Materials The data used to support the findings of this study.
